# Assessing fidelity to Critical Time Intervention (CTI) for homeless-experienced persons

**DOI:** 10.1186/s12913-026-14939-8

**Published:** 2026-06-16

**Authors:** Camilla Cummings, Maharshi Rawal, Gracielle Tan, Erin Finley, Kristina M. Cordasco, Daniel Herman, Lisa Edwards, Sonya Gabrielian

**Affiliations:** 1 VA Greater Los Angeles Healthcare System, Los Angeles, CA USA; 2 Departments of Medicine and Psychiatry & Behavioral Sciences, Joe R. and Teresa Lozano Long School of Medicine, University of Texas Health, San Antonio, TX USA; 3 Department of Medicine, David Geffen School of Medicine, University of California, Los Angeles, CA USA; 4 Silberman School of Social Work, Hunter College, The City University of New York, New York, NY USA; 5 Center for the Advancement of Critical Time Intervention (CACTI), New York, NY USA; 6 Jonathan and Karin Fielding School of Public Health, University of California, Los Angeles, CA USA; 7 Department of Psychiatry and Biobehavioral Sciences, David Geffen School of Medicine, University of California, Los Angeles, CA USA

**Keywords:** Fidelity assessment, Pragmatic trials, Behavioral health intervention

## Abstract

**Background:**

It is challenging and time-intensive to assess fidelity to complex evidence-based interventions in the homeless service sector. We describe a multi-component procedure to assess fidelity to Critical Time Intervention (CTI)—an evidence-based, time-limited case management practice for homeless-experienced persons undergoing housing transitions—developed for a large-scale pragmatic trial conducted with homeless-experienced Veterans.

**Methods:**

Using literature review, expert consultation, and pilot testing, we developed a pragmatic, scalable CTI fidelity assessment procedure. We integrated data from: a CTI implementation self-assessment designed to enhance provider practice; 90-minute videoconferences with case managers to collaboratively review charts from ≥2 randomly selected “exemplar cases” assessed for CTI’s core components; and field notes from case manager narratives during these videoconferences. At 12- and 18-month timepoints after CTI implementation began with 17 case managers across 15 homeless service agencies, we employed this procedure to assess fidelity to CTI. We used field notes to contextualize differences between self-assessment and expert-rated findings.

**Results:**

All case managers self-assessed their CTI practice as well- or ideally-implemented. Expert-rated assessments suggested that all case managers had limited fidelity to at least one of CTI’s core components; 4 agencies had inadequate fidelity to the overall practice. Field notes provided explanations for disparate results between the self- and expert-rated assessments, including skill deficits, staff turnover (resulting in limited understanding of CTI’s core components), and agency mandates (e.g. for case management visit frequency that were misaligned with CTI).

**Conclusions:**

The effectiveness of CTI is contingent on adherence to its core components. Streamlined approaches to fidelity assessment across EBPs in the homeless service sector is important for pragmatic, large-scale implementation efforts. The integration of self-assessment and expert-rated assessments can be applied to other multifaceted EBPs in the homeless service sector. Supplementing fidelity assessments with field notes may be useful to contextualize disparate findings between self- and expert-rated assessments, and to shape feedback to providers that enhances practice fidelity and improves housing outcomes.

**Trial registration:**

This project was registered with ClinicalTrials.gov as “Implementing and sustaining Critical Time Intervention in case management programs for homeless-experienced Veterans.” Trial registration NCT05312229, registered 4/4/2022.

**Supplementary information:**

The online version contains supplementary material available at 10.1186/s12913-026-14939-8.

## Introduction

Despite massive federal and state-level investments to address homelessness [1], there are more people experiencing homelessness in the United States than at any time since recording began in 2007 [2]. As such, there is a critical need to implement evidence-based practices (EBPs) that effectively and efficiently address homelessness [3]. Aligned with the complexity of this public health problem, these practices are often complex and multifactorial, ranging from prevention efforts, housing placement, financial subsidies, social services, and employment services, as well as health and behavioral health services [4]. In recent years, challenges associated with EBP implementation have garnered increased attention. These challenges include ensuring practice fidelity, selecting data-driven strategies to support the adoption and sustainment of effective practices, and de-implementing outdated practices when necessary [5–7].

It is well established that achieving intended outcomes for any EBP is contingent on adherence to core components of that practice [8, 9]. Despite calls for the application of implementation approaches to improve housing and health among persons experiencing homelessness, there is a paucity of research on the adoption and fidelity of EBPs in the homeless service sector (defined here as the wide range of organizations and providers assisting homeless persons through housing assistance, healthcare, and social services) [10]. Understanding the tension between fidelity to core components of EBPs and adaptations to local conditions is critically important, particularly within the context of homeless service providers, which often necessitate practice tailoring to fit geographic regions, housing markets, political and service contexts, as well as the diverse array of service providers providing care (considering funding mechanisms, organizational factors, staffing, and more) [11]. These complex contextual and organizational factors make it challenging, time-intensive, and costly to implement and assess the fidelity of multi-component behavioral health EBPs in the homeless service sector.

Across service sectors, assessing fidelity to complex behavioral health interventions is complicated, onerous, and costly, particularly within large-scale pragmatic trials [12, 13]. Further, extant implementation and fidelity research often lacks detailed procedural descriptions of fidelity assessment methods [12, 13]. Typical fidelity assessment methods may include ethnography, interviews, detailed checklists, and comprehensive medical record review [12]. Given the complexity of multi-level behavioral health interventions, scholars often recommend using a combination of methods to measure fidelity, as well as data integration across sources [9, 12–14].

Despite these recommendations, there is a dearth of research examining fidelity to real-world implementation of EBPs within the homeless service sector. The limited literature on this topic largely surrounds Housing First, a widely implemented EBP that combines subsidies for permanent and independent housing with field-based case management. Across studies, there are inconsistencies in the measurement of fidelity to this intervention, including differences in which core components are chosen and how they are defined or interpreted [15–19]. Though methods employed for fidelity assessment vary across the literature, nearly all employ labor-intensive and costly methods [15–19], involving detailed site visits with multi-method ethnographic data collection, review of administrative data, and sampling of multiple groups (e.g., supervisors, front-line providers, and program participants). While this research highlights the value of integrating disparate data sources in fidelity assessment, there are few, if any, examples of viable fidelity measurement procedures that can be employed in pragmatic, large-scale EBP implementation efforts in homeless service settings.

In 2020, the Department of Veterans Affairs (VA) Quality Enhancement and Research Initiative (QUERI) [20] funded a type 3 hybrid implementation-effectiveness trial [21] to assess the implementation of Critical Time Intervention (CTI)–a complex and structured case management EBP aimed to support vulnerable individuals during periods of transition–in 32 diverse community-based agencies across the nation that serve homeless-experienced Veterans undergoing housing transitions (e.g., unsheltered homelessness or transitional housing to independent housing) [22]. The Housing Transitions QUERI (HT QUERI) examines the impacts of evidence-based implementation strategies [23, 24] to support CTI adoption and sustainability. In HT QUERI, fidelity to the intervention was assessed as a primary implementation outcome, and as a measure of sustainment, at 12- and 18- months, respectively, after implementation launched. We developed a multi-method assessment procedure to assess fidelity and sustainment across diverse community-based settings. Our assessment of fidelity and sustainment was intended to guide and improve CTI implementation at the agency level and was also considered a key outcome in evaluating strategies used to support CTI implementation. The primary aims of this paper are to present the development of a pragmatic CTI fidelity assessment procedure; to describe the procedure; and to comment on its feasibility and use within a large pragmatic trial. To address these aims, we present illustrative examples from our fidelity assessment data and provide recommendations for ways this fidelity assessment approach can be applied to implementation initiatives of other EBPs in the homeless service or behavioral health sector.

## Methods

### Setting

In March 2019, VA awarded $30 million to 128 community-based homeless service agencies (coined “Grant and Per Diem (GPD)” sites) across the United States to provide 6 months of case management for homeless-experienced Veterans transitioning to independent living and not otherwise receiving case management (called the GPD case management “aftercare” program). The HT QUERI trial is described elsewhere [22]; in short, it implemented – between January 2022 through June 2024 – CTI across 32 aftercare programs, staggering implementation across 3 cohorts to compare the impacts of two implementation strategies on fidelity to CTI practice and clinical outcomes. All HT QUERI procedures were designated as non-research by the VA Central Institutional Review Board. Verbal informed consent was obtained from participants prior to participation in the fidelity assessment arm of the overarching trial.

### Evidence-based practice

**Fig. 1:**
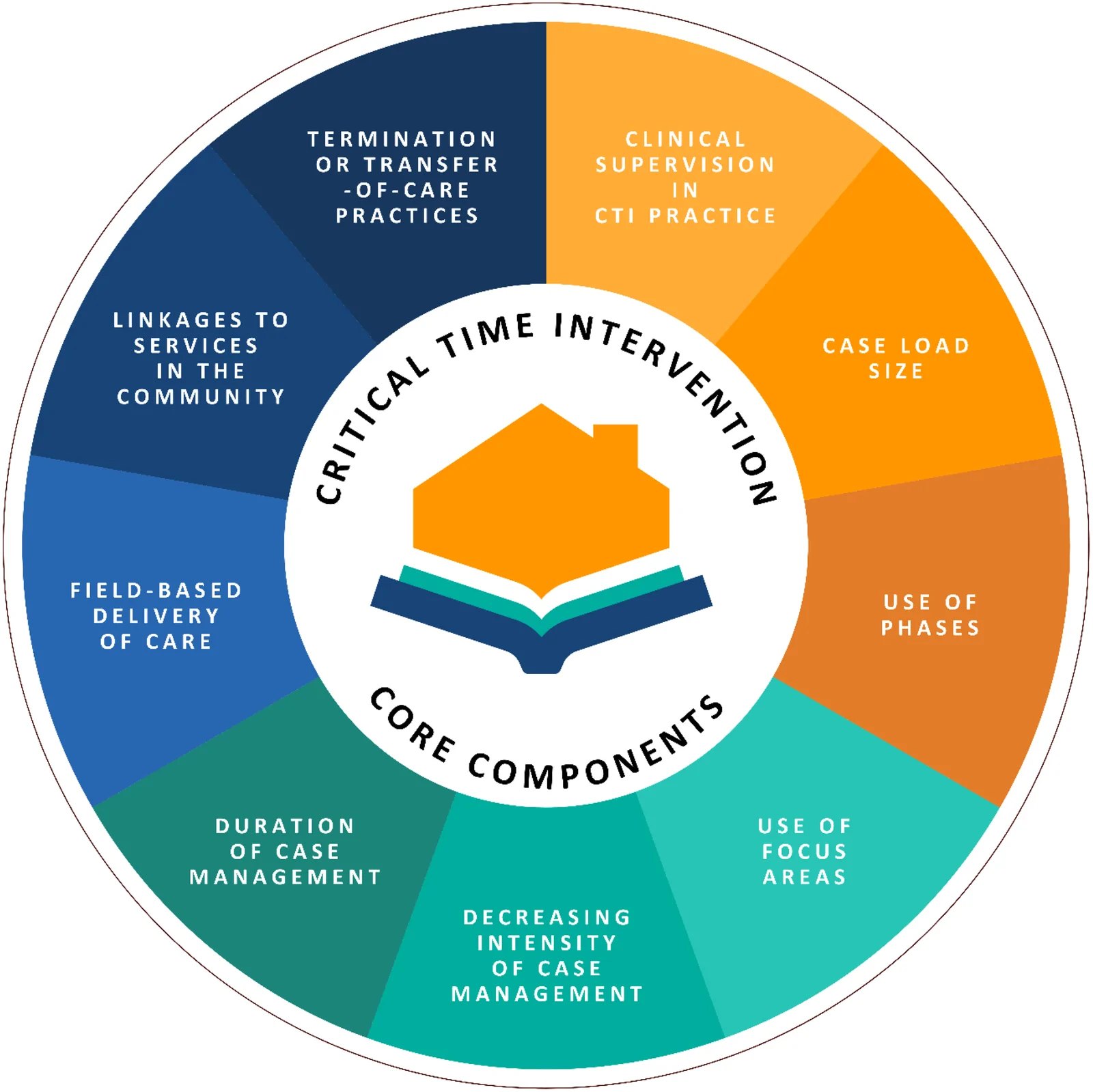
Critical Time Intervention (CTI) core components. Description: Fig. 1 presents an image depicting the 9 core components of Critical Time Intervention (CTI) with a footnote providing additional details about nuances related to the ways CTI core components were implemented in our pragmatic trial. *Note*. Weighted case load size is recommended at 20 clients, with different weights given to clients at each of the 3 phases of CTI. CTI is divided into 3 phases of decreasing case management intensity, each of at least 2 months in duration. Each of these phase has 1-3 recovery-oriented focus area goals. Duration of case management is time-limited, ranging from 6-9 months

CTI (Fig. 1) is a structured and time-limited case management EBP designed to reduce homelessness and adverse outcomes among vulnerable populations by enhancing continuity of care during challenging life transitions (e.g., moving from institutional settings to community living, homelessness to permanent housing) [25, 26]. A systematic review found robust evidence that CTI improves housing outcomes, continuity of care, and social service engagement, with more mixed results in terms of its impact on health service use, mental health, substance use, social support, and quality of life [27]. CTI is one of few EBPs for individuals experiencing homelessness [28–30]. The core components of CTI include time-limited service delivery, case management delivered in three distinct phases with goals for each phase and a tapering of service intensity across the phases, weekly supervision with a masters-or higher-level clinician trained in CTI, small caseloads, field-based service delivery, harm reduction philosophy, as well as a focus on making linkages to community-based services and social supports that will remain after the intervention period ends [25]. Despite strong evidence from randomized controlled trials (RCTs) that CTI is effective, infrequent and complex fidelity assessment procedures limit the ability to implement and disseminate this practice, with adherence to its core components, in real-world settings [27].

### Conceptual framework

**Fig. 2:**
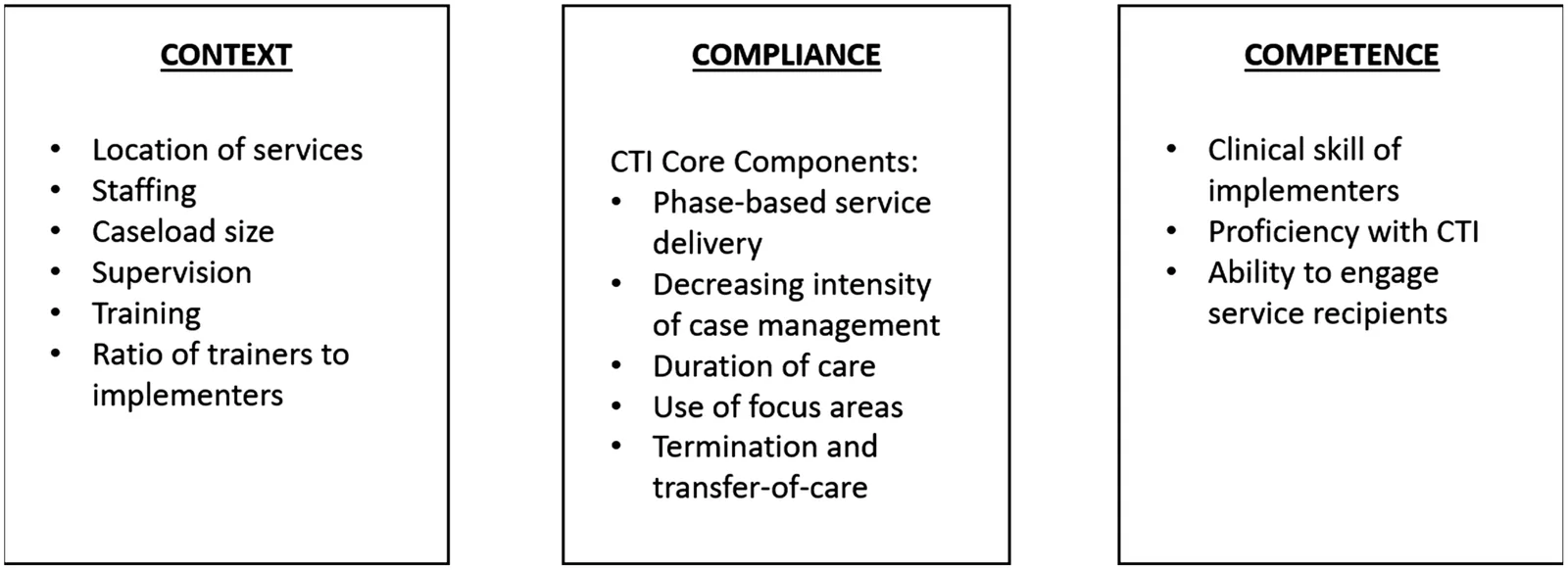
Dimensions of fidelity. Description: Fig. 2 presents the conceptual model of dimensions of fidelity including context (e.g., location of services, staffing, supervision), compliance (e.g., adherence to core components), and competence (e.g., clinical skill of implementers) fidelity

CTI fidelity is typically conceptualized in three dimensions, including context, compliance, and competency fidelity (Fig. 2) [8]. Context fidelity refers to precursors to implementation, including training, location of service provision, staffing, small caseload size, accessibility of resources and supervision, and reasonable ratios of trainers to implementers. Compliance fidelity refers to the core components of the intervention and the extent to which they are implemented. Competence fidelity includes the clinical skill of the implementers and the proficiency with which they engage participants and deliver the intervention.

### Development of the fidelity assessment procedure

Our multi-method fidelity assessment procedure was developed through review of the literature on CTI fidelity [31–33] consultation with national leaders in CTI (including the practice’s developers) [26], review and adaptation of established procedures and forms from other CTI fidelity assessment initiatives, as well as pilot testing and iterative adaptations. The pilot of CTI implementation was conducted over one year at four sites in Southern California, prior to the start of this 32-site trial [34]; the primary goals of the pilot were to develop training and technical assistance materials in preparation for the rapid scale up of the pragmatic trial. Fidelity assessment procedures were piloted at two of the four sites and iteratively refined through discussion with implementing case managers and an interdisciplinary evaluation team to develop the procedures used in this trial. We sought a practical, cost-effective method to assess context, compliance, and competence fidelity in this large national trial.

Specifically, we adapted established forms for CTI fidelity assessment—typically used to guide time-intensive, multi-method ethnography (often over two-day site visits) in well-resourced RCTs—for use in brief virtual visits [31–33]. We developed multi-method fidelity assessment methods that integrated data across three sources: provider self-assessment, expert-assessed virtual case review with standardized scoring, and field notes that captured narratives from the virtual case reviews. Expert-assessed case review procedures were piloted with one expert in separate one-hour videoconference meetings with two case managers from different agencies involved in the implementation pilot to assess the feasibility and acceptability of the procedures, virtual format, and time. Subsequent iterative adaptations were made to the case review guide, including details about clinical information to gather on each case, and ways to structure questions about visit modalities. Structured debrief sessions with case managers who participated in the pilot case review videoconferences suggested the procedures were feasible and acceptable, although one hour was not sufficient to complete the review and videoconferences were lengthened to 90 minutes. An additional notetaker was added to improve the quality of field notes and ensure reliability of case review data.

### Fidelity assessment procedure

HT QUERI recently completed implementation of CTI in 32 aftercare programs across three successive implementation cohorts, and we present data from the first two cohorts (21 sites) which occurred from January – September 2022 and from October 2022 – June 2023. Below, we describe a multi-component assessment procedure that was administered at 12 months post-implementation to measure fidelity to CTI; the same procedure was conducted again at 18 months to assess sustainment to CTI. Recruitment to participate in our data collection (i.e., self-assessment and expert-rated assessment) involved templated email outreach to all 21 participating sites, of which 15 sites completed at least one component of data collection. All 21 sites were recruited at the 12-month timepoint and only 16 of those were recruited at the 18-month timepoint due to funding nonrenewal. Unless sites explicitly declined, all sites received at least three email invitations to participate. At the 12-month timepoint, 15 of 21 sites participated (71%) in fidelity assessment. At the 18-month timepoint, 5 out of 16 sites participated (31%). As of 9/1/2024, encompassing both time points, we solicited participation from 30 unique case managers across the 21 agencies; 17 case managers (56%) across 15 agencies agreed to participate in both components of data collection.

### Self-assessment

The quantitative self-assessment (Additional File 1) employed a 5-point, Likert-type self-report scale (1 = never or rarely to 5 = always) comprised of 32 items assessing different dimensions of CTI practice; this scale—typically used to inform continuous self-improvement of CTI implementation by providers, as opposed to formal fidelity assessment—was administered via a web-based platform. The instrument assessed domains across context and compliance fidelity including time-limited service provision, phased delivery, focus areas per phase, caseload size, aspects of supervision, tapering of intensity, absence of early termination, aspects of care across three distinct phases (e.g., field-based service delivery, linkages to community supports, inclusion of social support), adherence to core philosophical principles of CTI (e.g., strength-based, person-centered, harm reduction), and completion of relevant documentation.

Using methods employed when this form is used for self-improvement [31], we scored the self-assessment by calculating the mean and standard deviation of scores across items. Scores were also averaged across items by domain to enable identification of higher versus lower fidelity CTI domains. The overall average was interpreted within a standardized rubric established by CTI’s developers in which 1-0.1.4 was “not implemented,” 1.5–2.4 was “poorly implemented,” 2.5–3.4 was “adequately implemented,” 3.5–4.4 was “well implemented,” and 4.5–5.0 was “ideally implemented.” Scores enabled comparison across providers and were combined to produce an average by agency (by averaging scores across providers in individual programs) for cross-agency comparison. Individual items were also averaged across providers and agencies to identify domains of CTI practice with higher versus lower implementation fidelity.

### Expert-rated assessment

We engaged front-line implementers (i.e., case managers) in expert-rated fidelity assessments, which involved: (1) the identification of exemplar cases (i.e., average or typical cases); (2) semi-structured review of exemplar cases via synchronous videoconference; and (3) standardized scoring of data from the exemplar case review. Expert assessors (C.C. and G.T.) were clinicians who were trained in CTI and engaged in the implementation initiative. Providers implementing CTI (i.e., case management staff across homeless service agencies) submitted caseload rosters that included demographic information and program entry and exit dates. These rosters were reviewed to identify 5 exemplar cases per provider, seeking a range of duration of case management engagement. As CTI encompasses 3 phases (here, 2 months each), we sought cases that completed all 3 phases (i.e., entry > 6 months prior to fidelity assessment with a program exit or discharge) if possible, or cases actively engaged in each of the 3 phases. We excluded cases with program entry < 2 months prior to fidelity assessment and, when applicable, included aberrant cases with early or late discharges (i.e., before or after the 6-month case management period).

Each case manager engaged in a 90-minute videoconference in which documentation and case management records of exemplar cases selected from the 5 identified were reviewed collaboratively; expert assessors recorded data in a semi-structured template (Additional File 2). On average, 2–3 cases were reviewed per case manager. The template gathered information across domains of context, compliance, and competence fidelity including supervision received, case demographic information, the episode of care (i.e., referral source, housing status, etc.), and a detailed review of service provision across the 3 phases of CTI (i.e., focus areas identified, dates of contact, modality, location of encounter, providers or support present, linkages made, focus areas achieved, transfer-of care procedures, etc.). These videoconferences were not recorded due to the discussion of protected health information (PHI); thus, two members of the assessment team participated in fidelity assessment procedures, with one serving as the primary assessor and another serving as a notetaker. No PHI was included in notes.

Primary assessors and notetakers participated in consensus meetings directly after each videoconference assessment to rectify discrepancies in data gathered (e.g., accuracy of clinical encounters, interpretations of ambiguous data) and finalize the templated exemplar case summary. Then, data from the final exemplar case summary was examined to complete a 10-domain standardized scoring tool (Additional File 3) adapted from and mirroring the domains captured in the self-assessment tool (i.e., domains of CTI practice). The assessor-rated fidelity scoring tool rated CTI practice domains on a 3-point Likert-type scale (1 = never to 3 = always). Qualitative descriptors associated with numerical scores were created to further standardize scoring. Ratings were discussed among two or more expert assessors until consensus was obtained. Assessor-rated assessment and standardized scoring enabled us to dichotomize sites as having “adequate” or “inadequate” CTI fidelity, based on having a majority (≥6) of domains or fewer, respectively, described as adequate. Sites were not scored when they reported no CTI clients on their caseload and/or when clinical documentation was not available for assessment (e.g., inadequate record-keeping, records stored off-site or otherwise inaccessible). Of note, when sites were not scored, they were excluded from this analysis, regardless of results of case managers’ self- assessments. Additional file 4 compares the extent to which CTI’s core components were measured across the self- and expert-rated fidelity assessments.

### Field notes

Detailed field notes [35] were taken by the primary assessor and notetaker within the context of the expert-rated fidelity assessment synchronous videoconferences. In addition to the standardized data collection as noted above, additional data emerged organically, encompassing context and competence fidelity. Field note data included geographic, organizational, and staff-related information that affected intervention delivery, idiosyncrasies related to documentation procedures, specific content of encounters, and intangible or qualitative information characterizing implementers’ clinical skill, proficiency of CTI, and client engagement skills. Field notes were recorded alongside exemplar case review data in the structured case review template [35]. These data were thus integrated with expert-rated assessment data for which there was a structured debriefing after each 90-minute session between the primary assessor and notetaker to attain consensus on any discrepancies. Field note data were analyzed utilizing principles of rapid qualitative methods (i.e., structured template summaries and matrix analyses) [36] and reviewed with a lens towards contextualizing differences between fidelity self-assessment and expert-rated assessment findings, as well as to provide narrative feedback to agency staff that could aid quality improvement.

### Feedback

We collated fidelity assessment data into agency-level feedback reports intended to iteratively improve CTI implementation and practice. Self-assessment data were presented with domain-level scores and a summary score with the interpretive range. Expert-rated assessment data were distilled to 9 total domains of fidelity, including clinical supervision in CTI practice, case load size, use of phases, use of focus areas, decreasing intensity of case management, duration of case management (condensed to include time-limited to 6 months and no early discharges), field-based delivery of care, linkages to services in the community, and termination or transfer-of-care practices. Individualized site-level reports (template provided in Additional File 5) included a synthesis of fidelity to CTI’s core components–integrating data across all data sources–and provided recommendations to support ongoing quality improvement efforts to enhance fidelity.

## Results

### Results of fidelity assessment

Results of the self- and expert-rated assessments are presented in Table 1. As depicted, 23 case managers (22 unique, 1 that participated at both the 12- and 18-month time-points) completed at least one assessment; 5 participants were excluded due to non-completion of one data collection arm. Only participants who completed both the self- and expert-rated assessments (*n* = 17) are included in our analytic comparison. All 17 participants self-assessed themselves as implementing CTI “well” (3.5–4.4) or “ideally” (4.5–5.0). This contrasted with expert-rated fidelity scores; of the 17 case managers who participated in both assessments, 12 provided adequate data to be assessed and dichotomized into adequate vs. inadequate categories. A third of participants could not be scored by the expert assessors (6; 35%) due to inadequate documentation or not having clients with whom they engaged in CTI and were thus excluded from analyses. Notably, these 6 participants self-assessed themselves as implementing CTI “well” or “ideally”. Nine of the 12 scorable sites (75%) obtained ≥ 6 out of 9 core components and were assessed as implementing CTI with adequate fidelity. Four sites (33%) obtained ≤ 5 out of 9 core components and were assessed as inadequately implementing CTI.

**Table 1:** Self- and expert-rated fidelity assessment scores

Site and Case Manager	Self-Assessment Rating	Expert-Assessment Rating
**12-month ratings**		
Site 1	3.5	Unable to score
Site 2	4.0	2.3
Site 3, Case Manager 1	4.8	2.1
Site 3, Case Manager 2	4.0	2.1
Site 4, Case Manager 1	4.5	2.4
Site 4, Case Manager 2	4.5	1.8
Site 5, Case Manager 1	4.1	2.9
Site 6	4.1	2.5
Site 7, Case Manager 1	4.1	2.6
Site 8, Case Manager 1	4.3	Unable to score
Site 8, Case Manager 2	3.8	Not assessed
Site 9	Not assessed	2.5
Site 10	4.5	2.8
Site 11	3.9	Unable to score
Site 12	4.2	2.3
Site 13, Case Manager 1	4.0	Unable to score
Site 14	3.6	Unable to score
Site 15	4.15	Not assessed
**18-month ratings**		
Site 3, Case Manager 3	Not assessed	1.9
Site 5, Case Manager 1	4.2	2.2
Site 7, Case Manager 2	4.6	Unable to score
Site 8, Case Manager 3	Not assessed	1.8
Site 13, Case Manager 2	4.2	2.0

Note. Ratings were interpreted as follows: 1.0–1.4 = not implemented, 1.5–2.4 = poorly implemented, 2.5–3.4 = adequately implemented, 3.5–4.4 = well implemented, and 4.5–5.0 = ideally implemented

Sites listed without specifying case manager number had only one case manager participant in assessment procedures

## Illustrative example

To illustrate the multi-method fidelity assessment procedure and its application, we describe findings from two front-line case managers within one agency; both participants completed self-assessment and expert-rated data collection at the 12-month timepoint. Both participants self-assessed their fidelity to CTI practice as “ideally implemented,” each scoring 4.5 out of 5. However, in the expert-rated assessment, both participants were rated as having “poorly implemented” CTI (case manager 1 = 2.4; case manager 2 = 1.8). The agency’s implementation of CTI overall was deemed as “inadequate,” with only 1 out of the 9 domains of practice scored as meeting standards. Specific illustration of fidelity to CTI’s core components (which are underlined) is detailed below.

### Adherence to practice core components

Expert-rater assessments found that case managers were not delivering field-based services, despite ranking themselves highly in this domain on self-assessment. Field note narratives revealed that the geographic context, which included many rural communities, made it prohibitively expensive and time-consuming to provide face-to-face care in the community; the organization did not have the resources to overcome these barriers, precluding the ability to provide field-based service delivery (that is, instead they relied on telephone or clinic-based care). Additionally, expert-rated scores found case managers did not taper service intensity over time, and one case manager identified many focus areas for each client far beyond the scope of case management.

Field notes enabled the identification of several contextual barriers to CTI fidelity, including the fact that agency leadership required a certain number of contacts with each client per month, which precluded the tapering of service intensity or frequency over time as outlined in the CTI model. Regarding the use of phases and focus areas, the two case managers had not used these components of CTI and struggled to define them, which contributed to their inflated self-assessment scores despite not using these components in their practice. Field notes described limited case management experience in these participants; they were entry-level staff in a challenging organizational context with frequent staff turnover, limited organizational support and supervision, little to no infrastructure for oversight and remediation of clinical skills, and poor institutional and clinical continuity. Data were synthesized and presented to the site within the fidelity feedback report. For example, links to the CTI manual and other resources were provided in the recommendation to identify only 1–3 focus areas, which might enable case managers to reduce the intensity of care over time.

## Discussion

The effectiveness of CTI is contingent on adherence to its core components. Streamlined approaches to fidelity assessment are important for large-scale implementation efforts, particularly in the homeless service sector, where high-fidelity EBP implementation may be especially challenging. We describe the development and use of a pragmatic, multi-component fidelity assessment procedure that integrated provider self-assessment data with brief and virtual expert-rated review. This procedure offers a straight-forward and low-burden method to elicit rich data for both research and quality improvement and may have utility for other large pragmatic implementation efforts of EBPs in homeless service and other low-resourced settings; however, we note that engaging case managers in voluntary participation in this and other fidelity assessment procedures is challenging.

HT QUERI, in performing the largest pragmatic implementation-focused trial of CTI to date, has encountered significant challenge in assessing practice fidelity across diverse agencies implementing this complex EBP. Expert ratings and ethnographic, narrative, or qualitative data are invaluable in assessing context and competence fidelity; however, it is not feasible to conduct multi-day in-person site visits to the number of sites participating in pragmatic trials of this scale. Moreover, as Bond and Drake [37] identify within the broader field of fidelity measurement, community-based agencies use widely different clinical documentation practices (e.g., paper files kept on site, a range of electronic health record platforms, or use of city or county-level databases like Homeless Management Information System (HMIS)); this variation, in combination with data protection regulations, obstruct analysis of administrative data. Although some case-level data were provided via caseload rosters, the structured data available in this study were limited, and assessments of competence and context fidelity relied upon field note narratives alongside expert review of clinical documentation [38].

Assessing clinical skill requires clinical judgment and observational methods, necessitating labor-intensive evaluation plans. Our multi-method procedure aimed to strike a balance of feasibility and rigor by employing observational methods and pragmatic analysis of client-level data; this process enabled assessment of competence and context fidelity without the cost, time, or labor burden of multi-day site visits. Although self-assessments (e.g., fidelity checklists) are easy to administer and low-cost, there are well-established concerns about the bias and validity of such methods [37, 39]. However, triangulating self- and expert-rated assessments enabled the identification of areas in which case managers demonstrated poor understanding of CTI core components. Having multiple sources of data also facilitated more fine-grain identification of implementation barriers. Of note, we had equivalent participation rates across self- and expert-rated assessments, despite differences in the time and effort required for participation, which suggests the 90-minute virtual videoconference was feasible from a participant perspective. Given the feasibility of this multi-method fidelity assessment procedure, we anticipate it to be of value for use within the homeless service sector, where applicable EBPs are inherently multifaceted and complex.

We note that our multi-method fidelity assessment procedure produced consistently divergent results across sources (i.e., case managers persistently self-assessed as implementing with higher fidelity than expert-rated assessments). Importantly, the self-assessment we adapted was designed for training, implementation, and technical assistance and therefore was not designed or validated as a fidelity assessment tool. The discrepancies found in our trial contrast findings in the overarching fidelity assessment literature wherein self- and expert-rated assessments are generally reliable; however, many practices in which self-assessments are used to capture fidelity employ methods specifically designed for fidelity measurement (as opposed to the tool used in our study, which was designed to improve practice quality as part of training and technical assessment) [40–42]. Differences in the self-assessment ratings could be related to a poorer understanding of the intervention among front-line implementers (as opposed to intervention experts) or rating domains that are not behaviorally-anchored or are more open to subjective interpretation (e.g., “Case managers work with clients to identify existing formal and informal community resources and supports.”) Developing a reliable self-assessment tool for CTI is an important future direction. Our data suggest the self-assessment holds value and future improvements might include adding descriptive anchors associated with item-level ratings, or the use of qualitative feedback per item or section to enable context for poorly implemented components which may reduce inflation of self-assessment scores. Increasing the reliability of the self-assessment tool and integrating open-ended response options to provide context for self-ratings may support streamlining and quality improvement of this method.

Nevertheless, these discrepancies found between our self- and expert-assessments underscore the importance of assessing fidelity in multiple ways, particularly during method development, and aligns with the literature recommending data or methods triangulation in fidelity assessments [12, 37, 38]. Relying on a single data source introduces significant bias or skew and limits the ability to assess all three conceptual domains of fidelity (i.e., compliance, context, and competence) [37]. Here, self-assessment scores alone would have resulted in an inflated appraisal of intervention fidelity and would not have yielded helpful data to guide iterative improvement or implementation. Conversely, though utilizing only expert-rated assessment scores may have provided a more realistic view of implementation, recommendations for improvement given to case managers may have been unrealistic or not feasible within the specific ecological context (e.g., field notes provided context indicating recommendations to increase field-based care delivery would be inappropriate). Thus, increasing the reliability of self-report measures (e.g., by using anchors to guide answer responses) is especially important for diverse, pragmatic implementation settings.

Integrating data across self- and expert-rated assessments enabled the identification of discrepant scores and areas of concern (e.g., case managers’ self-assessments indicated faithful identification of focus areas, yet expert reviews revealed focus areas were inappropriate or too many were chosen to be feasible). A meaningful subgroup of participants was excluded from analyses because they could not be scored by expert assessors due to inadequate documentation or no current clients with whom they were implementing CTI. We considered these participants’ data to be insufficient for assessing fidelity to the practice, either because they were not attempting to implement it at all or there were insufficient grounds on which to assess their implementation. We do not know why these participants self-assessed their fidelity so highly. It is possible they misconstrued assessors as related to funding bodies (though this was discussed in the informed consent) and were thus influenced by social desirability.

The use of field notes also provided critical data to reconcile divergent findings between self- and expert-rater assessments, identify case manager knowledge deficits regarding the intervention itself, and inform the development of pragmatic and feasible recommendations for quality improvement (e.g., providing direct links to templates, handouts, and training materials to improve the identification of appropriate focus areas). Integrating the field notes into the expert-rated assessment form may streamline the process and lower the burden for data collection, management, and analysis. Data integration across sources facilitated the development of a fidelity report which aimed to provide a summary of results, recommendations, and tailored resources to support implementers’ iterative improvement. Our procedure enabled the rapid development of provider- and -site-level performance feedback reports (see Additional File 5) which further serves as a quality improvement strategy to increase practice fidelity [43] and increases the method’s utility in pragmatic trials. Automating the integration of self- and expert-rated assessments and use of templated feedback reports are strategies to reduce the time, labor, and cost of fidelity assessment procedures similar to those described here.

Beyond assessing fidelity, our data demonstrate the tension between intervention fidelity and adaptation to setting and context [38, 44, 45]. For example, many agencies struggled to adhere to CTI’s core component of field-based care delivery. Field note narratives revealed complex service delivery environments wherein providers are low-paid, entry-level, often using their personal vehicles and sometimes without adequate transportation themselves. These complexities pose a question for deeper investigation around the extent to which outcomes are affected by a core component that is challenging to implement in a real-world setting; further, if change is needed, we question if it is more beneficial or feasible to adapt CTI delivery to the context (e.g., providing telephone or virtual care) or whether organizational and systemic factors require change to adopt CTI with fidelity. To date, no systematic evaluations regarding the efficacy of virtually-delivered CTI have been conducted, nor comparative studies examining non-inferiority with in-person care [26]. Our pragmatic trial may provide some guidance in evaluating the extent to which fidelity to core components impacts CTI’s outcomes in real-world practice, identifying systemic barriers to implementation, and perhaps in documenting the types of adaptations occurring on the ground. Future CTI research will need to grapple with the fidelity-adaptation tension, and we hope our contributions to improve fidelity assessment procedures will support such investigations.

Although we largely found this multi-method fidelity assessment procedure feasible and well-suited for pragmatic implementation, there are limitations worthy of note. As with other fidelity assessment methods described by Ginsburg and colleagues [12], we encountered missing data (e.g., due to differences in documentation across sites, data access issues, poor documentation practices, staff turnover, some sites did not have their funding renewed, attrition, lack of response, etc.) and unit of analysis issues (e.g., different staff received different “doses” of training and technical support, participation in some strands of data collection but not all, etc.). Particularly in high staff turnover environments—like our implementation context—any fidelity assessment procedure may be limited by missing data (e.g., from new staff unable to participate in assessments). Moreover, missing data are common in fidelity assessment [12] and many studies fail to report their missing data [46]. Slightly more than half of case managers agreed to participate in our fidelity assessment procedures and thus there is potentially a non-response bias that skews our fidelity findings. The small sample size in this study precluded greater examination of the psychometrics of both the self- and expert-rated fidelity assessments or the extent to which they are correlated. Future investigations of CTI fidelity assessment could build on this work by examining the self- and expert-rated fidelity assessments’ discriminative validity, sensitivity to change, and predictive validity on participant outcomes.[37]

Moreover, the CTI and broader fidelity assessment literature offers little guidance regarding how to weigh aspects of fidelity or core components, as the importance of each component’s contribution to outcomes is unclear [12]. Similarly, there are no best practice guidelines surrounding methods for synthesizing data sources in an overarching fidelity scheme or overall composite score. We chose to dichotomize into adequate versus inadequate categories based on the number of core components implemented well; however, the lack of standardization and differing measurement systems for evaluating fidelity across implementation research limits advancement in the field as a whole [38]. Although the broader evaluation team for our pragmatic trial has systematically collected data regarding real-time practice adaptations via periodic reflections [47], those data have not been integrated within our fidelity assessment procedures to date. Future investigations may integrate systematic assessment of adaptation as part of fidelity assessment methods. It is possible that our fidelity assessment procedures influenced internal validity (e.g., assessing core components or providing feedback might enhance practice fidelity); and in some ways, our procedures were designed to do that (e.g., self-assessment used as a self-monitoring tool, fidelity reports). The impact of feedback on implementation fidelity may be acceptable in pragmatic trials but may limit our ability to determine the extent to which specific training, technical assistance, or implementation strategies impact fidelity and outcomes. Of note, though it was beyond the scope of this investigation to assess the extent to which our fidelity feedback reports were utilized by front-line implementers, understanding how participants made use of feedback provided could be useful for future study.

## Conclusion

The multi-component assessment procedure outlined here offers a feasible method for assessing fidelity to complex behavioral health interventions across diverse implementation contexts, with promise for use within large-scale pragmatic implementation initiatives. Strengths of the method include its low costs, low burden to providers, flexibility to accommodate considerable differences in documentation and data access issues, inclusion of observational methods and clinical judgment enabling the assessment of competence and context fidelity, and use of data triangulation to produce more robust assessments of fidelity. We used these methods to identify barriers to faithful implementation and provide context-driven recommendations for practice improvement. Balancing feasibility with rigor, this fidelity assessment approach may be applicable for complex behavioral health EBPs within the homeless service sector more broadly. This method may benefit pragmatic implementation efforts by providing methodologic specificity, supporting standardization and best practices for the field of fidelity assessment, and maximizing the impact of EBPs in large-scale efforts to address homelessness.

## Electronic supplementary material

Below is the link to the electronic supplementary material.

Supplementary Material 1

Supplementary Material 2

Supplementary Material 3

Supplementary Material 4

Supplementary Material 5

## Data Availability

If the authors receive an e-mail request for access to data from this project, they will work to establish a Data Use Agreement (DUA). Pursuant to this DUA, they will create a limited dataset, excluding all participant identifiers, limiting use of the dataset, and prohibiting the recipient from identifying or re-identifying any individual whose data are included in the dataset.

## References

[1] HUD Public Affairs. U.S. Department of housing and Urban Development [Internet]. 2024, Jan, 29. Washington: U.S. Department. Press release No. 24-018, Biden-Harris Administration Awards $3.16 Billion in Homelessness Assistance Funding to Communities Nationwide [cited 2024 Jun 4]; [about 5 screens]. Available from: Biden-Harris Administration Awards $3.16 Billion in Homelessness Assistance Funding to Communities Nationwide | HUD.gov / U.S. Department of Housing and Urban Development (HUD).

[2] U.S. Department of Housing and Urban Development, Office of Community Planning and Development. The 2023 annual homelessness assessment report (AHAR) to Congress [Internet]. 2023. 2024 Jun 4. Washington: The U.S. Department of Housing and Urban Development]. Available from: 2023-AHAR-Part-1.pdf (huduser.gov).

[3] Olivet J, Paquette K, Hanson J, Bassuk E. The future of homeless services: an Introduction~!2009-08-18~!2009-09-28~!2010-03-22~!. TOHSPJ. 2010, Mar, 22;3(2):30–33. 10.2174/1874924001003020030.

[4] Hwang SW, Tolomiczenko G, Kouyoumdjian FG, Garner RE. Interventions to improve the Health of the HomelessA systematic review. Am J Prev Med. 2005, Nov, 1;29(4):311. 10.1016/j.amepre.2005.06.017. 16242595 10.1016/j.amepre.2005.06.017

[5] Sylvestre J, Kerman N. The evolution of housing first: perspectives of experts from the United States, Canada, and Europe. Eur J Homelessness _ Volume. 2024;18(1).

[6] Denvall V, Bejerholm U, Stylianides KC, Johanson S, Knutagård M. De-implementation: lessons to be learned when abandoning inappropriate homelessness interventions. IJOH. 2022, May, 26;2(2):152–68. 10.5206/ijoh.2022.2.13709.

[7] Kerman N, Stergiopoulos V. Addressing health needs in people with mental illness experiencing homelessness. Nat Ment Health. 2024, Apr;2(4):354–66. 10.1038/s44220-024-00218-0.

[8] Fixsen DL, Naoom SF, Blase KA, Friedman RM, Wallace F. Implementation research: a synthesis of the literature. Natl Implementation Res Network. 2005, Jan.

[9] Keith RE, Hopp FP, Subramanian U, Wiitala W, Lowery JC. Fidelity of implementation: development and testing of a measure. Implementation Sci. 2010, Dec;5(1):1–1. 10.1186/1748-5908-5-99. 10.1186/1748-5908-5-99 PMC3161382 21192817

[10] Casey R, Clark C, Smits P, Peters R. Application of implementation science for homeless interventions. Am J Public Health. 2013;103(S2):S183–84. 10.2105/AJPH.2013.301729. 10.2105/AJPH.2013.301729 PMC3969144 24148045

[11] O’Sullivan E, Nelson G, Aubry T, Estecahandy P, Laval C, Shinn M, et al. How social science can influence homelessness policy: experiences from Europe, Canada, and the United States-Part II: politics and policy change. Eur J Homelessness _ Volume. 2021;15(2).

[12] Ginsburg LR, Hoben M, Easterbrook A, Anderson RA, Estabrooks CA, Norton PG. Fidelity is not easy! Challenges and guidelines for assessing fidelity in complex interventions. Trials. 2021, May, 29;22(1):372. 10.1186/s13063-021-05322-5. 34051830 10.1186/s13063-021-05322-5 PMC8164256

[13] Toomey E, Hardeman W, Hankonen N, Byrne M, McSharry J, Matvienko-Sikar K, et al. Focusing on fidelity: narrative review and recommendations for improving intervention fidelity within trials of health behaviour change interventions. Health Phychol Behavioral Med. 2020, Jan, 1;8(1):132–51. 10.1080/21642850.2020.1738935. 10.1080/21642850.2020.1738935 PMC8114368 34040865

[14] Walton H, Spector A, Williamson M, Tombor I, Michie S. Developing quality fidelity and engagement measures for complex health interventions. Br J Health Psychol. 2020, Feb;25(1):39–60. 10.1111/bjhp.12394. 31693797 10.1111/bjhp.12394 PMC7004004

[15] Nelson G, Stefancic A, Rae J, Townley G, Tsemberis S, Macnaughton E, et al. Early implementation evaluation of a multi-site housing first intervention for homeless people with mental illness: a mixed methods approach. Evaluation Program Plann. 2014, Apr;43(43):16–26. 10.1016/j.evalprogplan.2013.10.004. 10.1016/j.evalprogplan.2013.10.004 24246161

[16] Gilmer TP, Stefancic A, Katz ML, Sklar M, Tsemberis S, Palinkas LA. Fidelity to the housing first model and effectiveness of permanent supported housing programs in California. Psychiatric Serv. 2014, Nov, 1;65(11):1311–17. 10.1176/appi.ps.201300447. 10.1176/appi.ps.201300447 25022911

[17] Kertesz SG, Austin EL, Holmes SK, DeRussy AJ, Van Deusen Lukas C, Pollio DE. Housing first on a large scale: Fidelity strengths and challenges in the VA’s HUD-VASH program. Psychological Serv. 2017, May;14(2):118. 10.1037/ser0000123. 10.1037/ser0000123 28481597

[18] Fletcher EH, Gabrielian S, Flynn AWP, Greenberg JM, Hovsepian S, Oberman RS, et al. Stakeholder perspectives on sustainment of housing first in a VA permanent supportive housing program. Health Serv Res. 2022, Apr;57(2):374–84. 10.1111/1475-6773.13904. 35238030 10.1111/1475-6773.13904 PMC8928030

[19] Choy-Brown M, Tiderington E, Tran Smith B, Padgett DK, Stefancic A. Strategies for sustaining fidelity: a multi-state qualitative analysis in housing first programs. Adm Policy Ment Health. 2021, Jan;48(1):36–45. 10.1007/s10488-020-01041-2. 32323216 10.1007/s10488-020-01041-2 PMC7581620

[20] McQueen L, Mittman BS, Demakis JG. Overview of the Veterans Health Administration (VHA) quality Enhancement research initiative (QUERI). J Am Med Inf Assoc. 2004, Sep, 1;11(5):339–43. 10.1197/jamia.M1499. 10.1197/jamia.M1499 PMC516239 15187071

[21] Curran GM, Landes SJ, McBain SA, Pyne JM, Smith JD, Fernandez ME, et al. Reflections on 10 years of effectiveness-implementation hybrid studies. Front Health Serv. 2022, Dec, 8;2:1053496. 10.3389/frhs.2022.1053496. 36925811 10.3389/frhs.2022.1053496 PMC10012680

[22] Gabrielian S, Finley EP, Ganz DA, Barnard JM, Jackson NJ, Montgomery AE, et al. Comparing two implementation strategies for implementing and sustaining a case management practice serving homeless-experienced veterans: a protocol for a type 3 hybrid cluster-randomized trial. Implementation Sci. 2022, Oct, 3;17(1):67. 10.1186/s13012-022-01236-1. 10.1186/s13012-022-01236-1 PMC9527738 36192785

[23] Kilbourne AM, Neumann MS, Pincus HA, Bauer MS, Stall R. Implementing evidence-based interventions in health care: application of the replicating effective programs framework. Implementation Sci. 2007, Dec;2(1):1–0. 10.1186/1748-5908-2-42. 10.1186/1748-5908-2-42 PMC2248206 18067681

[24] Kilbourne AM, Abraham KM, Goodrich DE, Bowersox NW, Almirall D, Lai Z, et al. Cluster randomized adaptive implementation trial comparing a standard versus enhanced implementation intervention to improve uptake of an effective re-engagement program for patients with serious mental illness. Implementation Sci. 2013, Dec;8(1):1–4. 10.1186/1748-5908-8-136. 10.1186/1748-5908-8-136 PMC3874628 24252648

[25] Herman D, Conover S, Felix A, Nakagawa A, Mills D. Critical time intervention: an empirically supported model for preventing homelessness in high risk groups. J Primary Prevent. 2007, Jul;28(3–4):295–312. 10.1007/s10935-007-0099-3. 10.1007/s10935-007-0099-3 17541827

[26] Herman DB, Susser ES, Conover SA, Conover S. Selected issues for the Next Decade. In: Critical time intervention: mobilizing supports for people during perilous transitions. Oxford University Press; 2024. p. 140–50.

[27] Manuel JI, Nizza M, Herman DB, Conover S, Esquivel L, Yuan Y, et al. Supporting vulnerable people during challenging transitions: a systematic review of critical time intervention. Adm Policy Ment Health. 2023, Oct;50(1):100–13. 10.1007/s10488-022-01224-z. 36229749 10.1007/s10488-022-01224-z PMC9832072

[28] Garcia C, Doran K, Kushel M. Homelessness and Health: factors, evidence, innovations that work, and Policy recommendations: an overview of factors and policy recommendations pertaining to homelessness and health. Health Aff (Millwood). 2024, Feb, 1;43(2):164–71. 10.1377/hlthaff.2023.01049. 38315930 10.1377/hlthaff.2023.01049

[29] Pottie K, Kendall CE, Aubry T, Magwood O, Andermann A, Salvalaggio G, et al. Clinical guideline for homeless and vulnerably housed people, and people with lived homelessness experience. Cmaj. 2020, Mar, 9;192(10):E240–54. 10.1503/cmaj.190777. 10.1503/cmaj.190777 PMC7062440 32152052

[30] Substance Abuse and Mental Health Services Administration. Behavioral Health services for people who are homeless. Treatment improvement protocol (TIP) series 55. HHS publication No. (SMA) 13-4734. 2013. Rockville, MD: Substance Abuse and Mental Health Services Administration.

[31] Conover S, Gilfillan S, Tomita A, Manuel J, Feeback R, Souvik P. Developing a fidelity measure for critical time intervention. Enhancing the impact of mental health services research. Unpublished.

[32] de Vet R, Lako DAM, Beijersbergen MD, van den Dries L, Conover S, van Hemert AM, et al. Critical time intervention for people leaving shelters in the Netherlands: assessing fidelity and exploring facilitators and barriers. Adm Policy Ment Health. 2017, Jan;44(1):67–80. 10.1007/s10488-015-0699-9. 26573154 10.1007/s10488-015-0699-9 PMC5225207

[33] Mascayano F, Alvarado R, Andrews HF, Jorquera MJ, Lovisi GM, Souza FMD, et al. Implementing the protocol of a pilot randomized controlled trial for the recovery-oriented intervention to people with psychoses in two Latin American cities. Cad Saúde Pública. 2019, May, 2;35(4):e00108018. 10.1590/0102-311x00108018. 10.1590/0102-311X00108018 PMC6688497 31066775

[34] Gabrielian S, Cordasco KM, Finley EP, Hoffmann LC, Harris T, Calderon RA, et al. Engaging stakeholders to inform national implementation of critical time intervention in a program serving homeless-experienced Veterans. Front Psychol. 2022, Dec, 14;13:1009467. 10.3389/fpsyg.2022.1009467. 36591052 10.3389/fpsyg.2022.1009467 PMC9795188

[35] Phillippi J, Lauderdale J. A guide to field notes for qualitative research: context and conversation. Qual Health Res. 2018, Feb;28(3):381–88. 10.1177/1049732317697102. 29298584 10.1177/1049732317697102

[36] Hamilton AB, Finley EP. Qualitative methods in implementation research: an introduction. Psychiatry Res. 2019, Oct, 1;280:112516. 10.1016/j.psychres.2019.112516. 31437661 10.1016/j.psychres.2019.112516 PMC7023962

[37] Bond GR, Drake RE. Assessing the fidelity of evidence-based practices: history and current status of a standardized measurement methodology. Adm Policy Ment Health. 2020, Nov;47(6):874–84. 10.1007/s10488-019-00991-6. 31691055 10.1007/s10488-019-00991-6

[38] Bond L, Simmons E, Sabbath EL. Measurement and assessment of fidelity and competence in nonspecialist-delivered, evidence-based behavioral and mental health interventions: a systematic review. SSM - Popul Health. 2022, Sep, 29;19:101249. 10.1016/j.ssmph.2022.101249. 36246092 10.1016/j.ssmph.2022.101249 PMC9563630

[39] Lee N, Cameron J. Differences in self and independent ratings on an organisational dual diagnosis capacity measure. Drug Alcohol Rev. 2009, Nov;28(6):682–84. 10.1111/j.1465-3362.2009.00116.x. 19930024 10.1111/j.1465-3362.2009.00116.x

[40] Couturier J, Kimber M, Barwick M, McVey G, Findlay S, Webb C, et al. Assessing fidelity to family-based treatment: an exploratory examination of expert, therapist, parent, and peer ratings. J Eat Disord. 2021, Dec;9(1):1–9. 10.1186/s40337-020-00366-5. 33446271 10.1186/s40337-020-00366-5 PMC7809847

[41] Rollins AL, McGrew JH, Kukla M, McGuire AB, Flanagan ME, Hunt MG, et al. Comparison of assertive community treatment fidelity assessment methods: reliability and validity. Adm Policy Ment Health. 2016, Mar;43(2):157–67. 10.1007/s10488-015-0641-1. 25721146 10.1007/s10488-015-0641-1

[42] Rollins AL, Kukla M, Salyers MP, McGrew JH, Flanagan ME, Leslie DL, et al. Comparing the costs and acceptability of three fidelity assessment methods for assertive community treatment. Adm Policy Ment Health. 2017, Sep;44(5):810–16. 10.1007/s10488-016-0785-7. 28054197 10.1007/s10488-016-0785-7

[43] Burns MK, Peters R, Noell GH. Using performance feedback to enhance implementation fidelity of the problem-solving team process. J Sch Psychol. 2008, Oct, 1;46(5):537–50. 10.1016/j.jsp.2008.04.001. 19083371 10.1016/j.jsp.2008.04.001

[44] Chambers DA, Glasgow RE, Stange KC. The dynamic sustainability framework: addressing the paradox of sustainment amid ongoing change. Implementation Sci. 2013, Dec;8(1):1–1. 10.1186/1748-5908-8-117. 10.1186/1748-5908-8-117 PMC3852739 24088228

[45] Reed JE, Howe C, Doyle C, Bell D. Simple rules for evidence translation in complex systems: a qualitative study. BMC Med. 2018, Dec;16(1):1–20. 10.1186/s12916-018-1076-9. 10.1186/s12916-018-1076-9 PMC6009041 29921274

[46] Walton H, Spector A, Tombor I, Michie S. Measures of fidelity of delivery of, and engagement with, complex, face-to-face health behaviour change interventions: a systematic review of measure quality. Br J Health Psychol. 2017, Nov;22(4):872–903. 10.1111/bjhp.12260. 28762607 10.1111/bjhp.12260 PMC5655766

[47] Finley EP, Huynh AK, Farmer MM, Bean-Mayberry B, Moin T, Oishi SM, et al. Periodic reflections: a method of guided discussions for documenting implementation phenomena. BMC Med Res Methodol. 2018, Dec;18(1):1–5. 10.1186/s12874-018-0610-y. 30482159 10.1186/s12874-018-0610-y PMC6258449

