# Supplementary material for: Assessing fidelity to Critical Time Intervention (CTI) for homeless-experienced persons
Source: BMC Health Serv Res. 2026 Jun 16;26:1185. doi: 10.1186/s12913-026-14939-8 (PMC13508192; doi:10.1186/s12913-026-14939-8)
Supplement: Supplementary file 1 [file 12913_2026_14939_MOESM1_ESM.docx]

**Additional File 1: Critical Time Intervention (CTI) Implementation Self-Assessment Form**

a. Description: Additional file 1 presents the Likert-type self-assessment measure administered to case management staff to assess their perception of their practice fidelity to CTI’s core components. This measure is included to support standardization and replicability of the multi-method fidelity assessment procedure.

**Critical Time Intervention (CTI) Implementation Self-Assessment Form**

| **Never or Rarely** | **Sometimes** | **About half of the time** | **Most of the time** | **Always** |
| --- | --- | --- | --- | --- |
| **1** | **2** | **3** | **4** | **5** |

| **CTI Core Components** | **Score**  (1-5) |
| --- | --- |
| **Time-Limited**   1. CTI lasts no longer than 6 months (from the date the Veteran started Phase 1). |  |
| **Three Phases**   2. Beginning after Pre-CTI, the intervention takes place in three phases, each phase lasting two months. |  |
| **Focused**   3. Case managers select 1-3 focus areas for each phase. |  |
| 4. All focus areas in each phase are selected from the list of predetermined CTI areas on the Phase Plan: benefits; employment; survival needs (e.g., food, clothing, furniture; childcare; transportation); education (child/adult); natural supports; budget management; health and mental health; children’s health and mental health; housing; legal concerns. |  |
| 5. Focus areas are based on their relevance to the Veteran’s long-term housing stability. |  |
| **Small Caseload Size**   6. Each case manager has no more than 20 weighted cases per month (using the Weighted Caseload Tracker). |  |
| **Weekly Team Supervision Meetings**   7. Supervision takes place as a team, consisting of the supervisor and more than one case manager. (For agencies with only one case manager, ‘team supervision’ is between the supervisor and case manager). |  |
| 8. Team supervision meetings are led by the supervisor, who is a clinician and has been trained in CTI. |  |
| 9. Team supervision meetings take place weekly. |  |
| 10. Supervision is used to reinforce practices consistent with the CTI model and to correct practices that are noted. |  |
| 11. During supervision sessions, the team discusses each Veteran in the context of his/her current phase in CTI at least once a month. |  |
| 12. Case managers give a case presentation at the supervision meeting for each new Veteran on their caseload. |  |
| 13. Some (~6-8) high priority Veterans are selected prior to each supervision meeting for in-depth discussion by the team. |  |
| 14. Supervisor monitors case managers’ documentation regularly to ensure high quality and timeliness. |  |
| 15. Supervisors and case managers identify relevant VA and community resources together and discuss resource linkages during supervision meetings. |  |
| **Decreasing Contact Over Three Phases**   16. As Veterans progress through Phases and become connected to resources*, case managers decrease frequency of contact**and shift to mediator/monitor role. |  |
| **No Early Termination**   17. CTI does not end earlier than before six months (from the date the Veteran started Phase 1). |  |
| **Phases of CTI** | |
| **Phase 1**   18. 18.Case managers gather Veteran information to enable a best fit between the Veteran and VA and community resources, e.g., his/her interests, skills, strengths, vulnerabilities, aspirations, and history, such as education, jobs, housing, and treatment. |  |
| 19. Case managers explore neighborhood with the Veteran in order to foster new community-based relationships and skills. |  |
| 20. Case managers have on average one contact per week with the Veteran and four of these contacts take place in the community (e.g., Veteran's apartment, service agency). |  |
| 21. Case managers actively link Veterans to formal and informal supports where needed and strengthen relationships with existing resources. |  |
| 22. Case managers work with Veterans to identify existing formal and informal community resources and supports. |  |
| **Phase 2**   23. Case managers have on average two contacts per month with the Veteran and at least 2 of these contacts take place in the community. |  |
| 24. Case managers mediate between the Veteran and his/her support network. |  |
| 25. Case managers assess the strength of linkages by monitoring and recording the Veteran’s interaction with support network. |  |
| **Phase 3**   26. Case managers have on average one contact per month with the Veteran and at least one of these contacts takes place in the community. |  |
| 27. Case managers ensure direct communication between different members of Veterans’ support network. |  |
| 28. Before the end of CTI, case managers have a Transfer-of-Care meeting or call with members of the Veteran’s support network with whom it is necessary to meet, e.g., the case managers should transfer care with case managers, but may not need to meet daycare providers. (The Veteran should be present if possible.) |  |
| 29. Before the end of CTI, case managers have a Closing Meeting with the Veteran to discuss his/her experience with CTI and relationship with case manager, expectations for the future, and long-term support network’s contact information. |  |
| **Case Managers’ Role** | |
| 30. Case managers use a strengths-based, person-centered approach that incorporates shared decision-making in their interactions with Veterans (e.g., relate to them in genuine ways, as about topics not related to treatment, normalize their feelings) |  |
| 31. Case managers take a harm-reduction approach to planning with Veterans, when applicable. |  |
| **Documentation** | |
| 32.A progress note is completed for each contact with the Veteran and/or member of his/her support network. |  |
| **Total of scores for items 1 through 32** |  |
| **Average CTI Implementation Score (total divided by 32)** |  |

*’Support network’ and ‘community resources’ both refer to ‘formal resources’ (e.g., non-VA providers, VA providers if healthcare eligible) and ‘informal resources’ (e.g., family members, friends, neighbors, landlord, spiritual communities)

**A ‘contact’ refers to a phone call, video conference, face-to-face meeting (office or community), text, secure message, or e-mail

| **Not**  **Implemented** | **Poorly**  **Implemented** | **Adequately Implemented** | **Well**  **Implemented** | **Ideally**  **Implemented** |
| --- | --- | --- | --- | --- |
| **1.0-1.4** | **1.5-2.4** | **2.5-3.4** | **3.5-4.4** | **4.5-5.0** |
