# Supplementary material for: Assessing fidelity to Critical Time Intervention (CTI) for homeless-experienced persons
Source: BMC Health Serv Res. 2026 Jun 16;26:1185. doi: 10.1186/s12913-026-14939-8 (PMC13508192; doi:10.1186/s12913-026-14939-8)
Supplement: Supplementary file 2 [file 12913_2026_14939_MOESM2_ESM.docx]

**Additional File 2: Critical Time Intervention (CTI) Expert-rated Exemplar Case Review Template**

a. Description: Additional file 2 presents the semi-structured exemplar case review template used by CTI expert fidelity assessors during the 90 minute fidelity assessment videoconference. This template is included to support standardization and replicability of the multi-method fidelity assessment procedure.

**Critical Time Intervention (CTI) Expert-rated Exemplar Case Review Template**

Site:

Case Manager:

Assessor:

Notetaker:

**Beginning of Interview:**

*Today, we have planned a video conference that is intended to assess how CTI is being used in your program. It is NOT meant to critique your service delivery or documentation processes, but rather will help us collect information will help us better implement CTI across settings in the future.*

*In preparation for this meeting, our staff reviewed the roster of Veterans you case managed and randomly selected Veterans on each case manager’s caseload in the past 6 months as examples. In our meeting today, we’ll be reviewing each of these cases one by one. I’ll ask you to review your documentation for the case and then I’ll ask some follow up questions.*

*The notes column is a space to include case manager narratives about reasons for their answers to structured questions, e.g., reason why certain modalities of visits were preferred, reasons why transitions were delayed.

# CASE 1

| **Question** | | **Answer** | | | | | | | | **Notes*** | |
| --- | --- | --- | --- | --- | --- | --- | --- | --- | --- | --- | --- |
| Case Manager Name | |  | | | | | | | |  | |
| Veteran Name | |  | | | | | | | |  | |
| Veteran ID: | | *(Age, gender, diagnoses, disability/impairments, identifying characteristics—mental health, substance use disorders)* | | | | | | | | | |
| Did you work directly with this Veteran in CTI? (yes/no) | | Transition of care:  Where did they transition from? | | | | | | | | Current Housing Status:  What is their current housing status and do they live alone? If no, who are they living with? | |
|  | |  | | | | | | | |  | |
| How frequently have you received supervision on this veteran’s case?  What degrees does this supervisor have? Was he/she trained in CTI? | | Frequency | | Supervisor’s degrees | | | | Supervisor trained in CTI?  (yes/no) | |  | |
|  |  |  | |  | | | |  | |  |  |
| **Phase 1** | | | | | | | | | | | |
| Date this phase actually started | |  | | | | | | | |  | |
| What were the focus areas for this phase? How does each area support this veteran's housing stability? | | Focus areas | | | How supports long-term housing stability? | | | | |  | |
|  |  |  | | |  | | | | |  |  |
|  |  |  | | |  | | | | |  |  |
|  |  |  | | |  | | | | |  |  |
| Please tell me the date of each contact you had (either with the veteran and/or with supports present) in this phase, the type of contact, where each meeting took place, and which supports were also present, if any | Date | | Type of contact  (face-to-face, video, phone, text, email) | | | Location, if face-to-face  (CTI office / Community) | Was the  veteran  present? (yes/no) | | Was support present?  (yes/no)  List all that apply: VA provider, non-VA provider, informal | | Were linkages made?  If so, what linkages?  (and space for any additional comments) |
|  |  | |  | | |  |  | |  | |  |
|  |  | |  | | |  |  | |  | |  |
|  |  | |  | | |  |  | |  | |  |
|  |  | |  | | |  |  | |  | |  |
|  |  | |  | | |  |  | |  | |  |
|  |  | |  | | |  |  | |  | |  |
|  |  | |  | | |  |  | |  | |  |
|  |  | |  | | |  |  | |  | |  |
|  |  | |  | | |  |  | |  | |  |
|  |  | |  | | |  |  | |  | |  |
|  |  | |  | | |  |  | |  | |  |
|  |  | |  | | |  |  | |  | |  |
|  |  | |  | | |  |  | |  | |  |
|  |  | |  | | |  |  | |  | |  |
|  |  | |  | | |  |  | |  | |  |
| Can we get a summary of the focus areas achieved and the successful linkages made during this phase? Did you include the Veterans’ existing social support if they were available? Did you support the Veteran in reconnecting with or cultivating new social supports? To what extent did you provide linkages to community-based resources outside of your program? | | | | | | | | | | | |

| **Phase 2** | | | | | | | | | |
| --- | --- | --- | --- | --- | --- | --- | --- | --- | --- |
| Date this phase actually started | |  | | | | | |  | |
| What were the focus areas for this phase? How does each area support this veteran's housing stability?" | | Focus areas | | How supports long-term housing stability? | | | |  | |
|  |  |  | |  | | | |  |  |
|  |  |  | |  | | | |  |  |
|  |  |  | |  | | | |  |  |
| Please tell me the date of each contact you had (either with the veteran and/or with supports present) in this phase, the type of contact, where each meeting took place, and which supports were also present, if any | Date | | Type of contact  (face-to-face, video, phone, text, email) | | Location, if face-to-face  (CTI office / Community) | Was the  veteran  present? (yes/no) | Was support present?  (yes/no)  List all that apply: VA provider, non-VA provider, informal | | Were linkages made?  If so, what linkages?  (and space for any additional comments) |
|  |  | |  | |  |  |  | |  |
|  |  | |  | |  |  |  | |  |
|  |  | |  | |  |  |  | |  |
|  |  | |  | |  |  |  | |  |
|  |  | |  | |  |  |  | |  |
|  |  | |  | |  |  |  | |  |
|  |  | |  | |  |  |  | |  |
|  |  | |  | |  |  |  | |  |
|  |  | |  | |  |  |  | |  |
|  |  | |  | |  |  |  | |  |
|  |  | |  | |  |  |  | |  |
|  |  | |  | |  |  |  | |  |
|  |  | |  | |  |  |  | |  |
|  |  | |  | |  |  |  | |  |
|  |  | |  | |  |  |  | |  |
| Can we get a summary of the focus areas achieved and the successful linkages made during this phase? Did you include the Veterans’ existing social support if they were available? Did you support the Veteran in reconnecting with or cultivating new social supports? To what extent did you provide linkages to community-based resources outside of your program? | | | | | | | | | |

| **Phase 3** | | | | | | | | | | | | | | | |
| --- | --- | --- | --- | --- | --- | --- | --- | --- | --- | --- | --- | --- | --- | --- | --- |
| Date this phase actually started | | |  | | | | | | | | | | |  | |
| Date this phase actually ended | | |  | | | | | | | | | | |  | |
| What were the focus areas for this phase? How does each area support this veteran's housing stability?" | | | Focus areas | | | How supports long-term housing stability? | | | | | | | |  | |
|  |  |  |  | | |  | | | | | | | |  |  |
|  |  |  |  | | |  | | | | | | | |  |  |
|  |  |  |  | | |  | | | | | | | |  |  |
| Please tell me the date of each contact you had (either with the veteran and/or with supports present) in this phase, the type of contact, where each meeting took place, and which supports were also present, if any | Date | | | Type of contact  (face-to-face, video, phone, text, email) | | | Location, if face-to-face  (CTI office / Community) | | Was the  veteran  present? (yes/no) | Was support present?  (yes/no)  List all that apply: VA provider, non-VA provider, informal | | | | | Were linkages made?  If so, what linkages?  (and space for any additional comments) |
|  |  | | |  | | |  | |  |  | | | | |  |
|  |  | | |  | | |  | |  |  | | | | |  |
|  |  | | |  | | |  | |  |  | | | | |  |
|  |  | | |  | | |  | |  |  | | | | |  |
|  |  | | |  | | |  | |  |  | | | | |  |
|  |  | | |  | | |  | |  |  | | | | |  |
|  |  | | |  | | |  | |  |  | | | | |  |
|  |  | | |  | | |  | |  |  | | | | |  |
|  |  | | |  | | |  | |  |  | | | | |  |
|  |  | | |  | | |  | |  |  | | | | |  |
|  |  | | |  | | |  | |  |  | | | | |  |
|  |  | | |  | | |  | |  |  | | | | |  |
|  |  | | |  | | |  | |  |  | | | | |  |
|  |  | | |  | | |  | |  |  | | | | |  |
|  |  | | |  | | |  | |  |  | | | | |  |
| Can we get a summary of the focus areas achieved and the successful linkages made during this phase? Did you include the Veterans’ existing social support if they were available? Did you support the Veteran in reconnecting with or cultivating new social supports? To what extent did you provide linkages to community-based resources outside of your program? | | | | | | | | | | | | | | | |
| Please tell me the date of Transfer-of-Care visit(s) with members of the veteran’s support network, the type of visit, which supports were present, and if the veteran was present | | Date | | | Type of visit | | | Which supports?  List all that apply: VA provider, non-VA provider, informal | | | Veteran present?  (yes/no) | |  | | |
|  |  |  | | |  | | |  | | |  | |  | | |
|  |  |  | | |  | | |  | | |  | |  |  |  |
|  |  |  | | |  | | |  | | |  | |  |  |  |
| Please tell me the date of Closing Meeting with the veteran ONLY and what was discussed | | Date | | | Did you discuss ALL of following?  1-veteran’s experience with CTI  2-relationship with case manager  3-expectations for the future  4-long-term support network’s contact information  (yes / no) | | | | | | | If not, which did you discuss?  List numbers for each item discussed |  | | |
|  |  |  | | |  | | | | | | |  |  | | |
| *(If the case is still open for more than 6 months prior to the date of interview)* Why was this case extended? | | | | | | | | | | | | | | | |
