# Supplementary material for: Assessing fidelity to Critical Time Intervention (CTI) for homeless-experienced persons
Source: BMC Health Serv Res. 2026 Jun 16;26:1185. doi: 10.1186/s12913-026-14939-8 (PMC13508192; doi:10.1186/s12913-026-14939-8)
Supplement: Supplementary file 3 [file 12913_2026_14939_MOESM3_ESM.docx]

**Additional File 3: Critical Time Intervention (CTI) Expert-Rated Sustainability and Fidelity Assessment Tool**

a. Description: Additional file 3 presents the scoring tool used by CTI expert fidelity assessors including the data source for each item and qualitative descriptors to support standardized scoring. This measure is included to support standardization and replicability of the multi-method fidelity assessment procedure.

**Critical Time Intervention (CTI) Expert-Rated Sustainability and Fidelity Assessment Tool**

First Scorer: NAME (X/XX/23)

Site:

This scale is used to make fidelity ratings of CTI’s core components for VA Grant and Per Diem-Case Management (GPD-CM) sites. It was adapted from the CTI Implementation Self-Assessment Form as well as fidelity scoring procedures developed by the Center for the Advancement of CTI (CACTI) and the Center for Urban Community Services (CUCS).

*All questions are rated on a 3-point Likert scale (1, 2, 3, with 1 being lowest and 3 being highest)*

| **CTI Core Components** | **Data Source** | **Score**  (1-3) | **Notes** |
| --- | --- | --- | --- |
| **Weekly team supervision** | | | |
| 1. The team has weekly team supervision meetings, led by the clinical supervisor, who is a psychiatrist, psychologist, master’s level social worker (MSW), or another master’s level clinician who is trained in CTI | Exemplar cases |  | - 3 = all three characteristics of supervision (weekly, masters-level or higher educational level, trained in CTI) - 2 = two characteristics - 1 = one or fewer characteristics |
| **Time-limited** | | | |
| 2. CTI lasts no longer than 6 months (from the date the Veteran started Phase 1) | Case roster |  | - Count all veterans with exit dates, regardless of exit reasons - 182 + 7 days (up to 189) - # of veterans with d/c date up to 189 days divided by total veterans with exit dates - Include: Other - Exclude: Deceased and Transitioned to different level of care - Cutoff/categories   - 3 = 67-100%   - 2 = 34-66%   - 1 = 0-33% |
| **No early discharges** | | | |
| 3. CTI did not end earlier than 6 months (from the date the Veteran started Phase 1) | Case roster |  | - Count all veterans with exit dates, regardless of exit reasons - 182 - 7 days (175 or more) - # of veterans with d/c date 175 or more days divided by total veterans with exit dates - Include: Other - Exclude: Deceased and Transitioned to different level of care - Cutoff/categories   - 3 = 67-100%   - 2 = 34-66%   - 1 = 0-33% |
| **Phased Intervention** | | | |
| 4. Beginning after Pre-CTI, the intervention takes place in 3 phases, each phase lasting 2 months | Exemplar cases |  | - ± 7 day leeway between phases - Always (3), Sometimes (2), Never (1) |
| **Focused** | | | |
| 5. Only one to three focus areas for each phase are selected from the areas specific to this site | Exemplar cases |  | - Always (3), Sometimes (2), Never (1) |
| **Small caseload size** | | | |
| 6. Each case manager has no more than 20 weighted cases* on his/her caseload | Case roster |  | - If site does not use phases, count by the two-month intervals, with months 1-2 being phase 1, 3-4 being phase 2, and 5-6 being phase 3 - Goal is <20 - Use the weighted caseload tracker - Dichotomous scoring (3 = yes; 1 = no) |
| **Community-based** | | | |
| 7. To what extent does the case manager provide community- or field-based meetings? | Exemplar cases |  | - Always (3), Sometimes (2), Never (1) |
| **Decreasing contacts** | | | |
| 8. To what extent does the case manager taper the intensity of the intervention or reduce contact over time? | Exemplar cases |  | - If site does not use phases, count by the two-month intervals, with months 1-2 being phase 1, 3-4 being phase 2, and 5-6 being phase 3 - Always (3), Sometimes (2), Never (1) |
| **Engaging existing supports** | | | |
| 9. To what extent does the case manager engage existing formal or informal supports? | Exemplar cases |  | - Exclude data/case if Veteran does not have existing supports (i.e., N/A) - Always (3), Sometimes (2), Never (1) |
| **Providing linkages** | | | |
| 10. To what extent does the case manager provide linkages or support in establishing formal or informal supports? | Exemplar cases |  | - Always (3), Sometimes (2), Never (1) |
| **Total score for items 1-10** | |  | |
| **Average score (total score divided by 10)** | |  | |
