# Supplementary material for: Assessing fidelity to Critical Time Intervention (CTI) for homeless-experienced persons
Source: BMC Health Serv Res. 2026 Jun 16;26:1185. doi: 10.1186/s12913-026-14939-8 (PMC13508192; doi:10.1186/s12913-026-14939-8)
Supplement: Supplementary file 4 [file 12913_2026_14939_MOESM4_ESM.docx]

**Additional File 4: Comparison of Self- vs. Expert-rated Fidelity Assessment on CTI Core Components**

a. Description: Additional file 4 presents a table comparing which CTI core components are assessed across the self- and expert-rated fidelity assessment measures. This table is included to inform readers about the overlap and differences in what is measured across data sources.

**Comparison of Self- vs. Expert-rated Fidelity Assessment on CTI Core Components**

| **CTI Core Components** | **Self-Assessment** | **Expert-rated Assessment** |
| --- | --- | --- |
| 1. Case manager philosophy (strengths-based, person-centered, harm reduction) | X |  |
| 2. Documentation | X |  |
| 3. Clinical supervision | X | X |
| 4. Time-limited (no longer than 6 months) | X | X |
| 5. No early discharge / termination | X | X |
| 6. 1-3 focus areas identified per phase | X | X |
| 7. Small caseload size | X | X |
| 8. Decreasing contact / intensity across phases | X | X |
| 9. Phased delivery | X | X |
| 10. Community-based service delivery | X | X |
| 11. Engage existing supports | X | X |
| 12. Provide linkages to formal and informal supports | X | X |
| 13. Termination and transfer-of-care | X |  |
