# Supplementary material for: Assessing fidelity to Critical Time Intervention (CTI) for homeless-experienced persons
Source: BMC Health Serv Res. 2026 Jun 16;26:1185. doi: 10.1186/s12913-026-14939-8 (PMC13508192; doi:10.1186/s12913-026-14939-8)
Supplement: Supplementary file 5 [file 12913_2026_14939_MOESM5_ESM.docx]

**Additional File 5: Critical Time Intervention (CTI) Fidelity Assessment Feedback Report Template**

a. Description: Additional file 5 presents a template outline of the fidelity assessment feedback reports given to participating sites. This template is provided to illustrate the ways expert-and self-assessment data were synthesized and presented for iterative quality improvement.

**Critical Time Intervention (CTI) Fidelity Assessment Feedback Report Template**

1. Background and introduction describing the CTI implementation initiative

[Insert text]

2. Description of the multi-method fidelity assessment procedure

[Insert text]

3. Self-Assessment Rating

| **Fidelity Domain** | | | | **Score** |
| --- | --- | --- | --- | --- |
| Role and function of case managers | | | | XX |
| CTI documentation practices | | | | XX |
| Clinical supervision in CTI practice | | | | XX |
| Duration of case management and termination practices | | | | XX |
| Core structural components of CTI (decreasing intensity of case management, use of phases, focus areas, case load size) | | | | XX |
| CTI Phase 1 (field-based service delivery, linkages to community supports, inclusion of social support) | | | | XX |
| CTI Phase 2 (field-based service delivery, linkages to community supports, inclusion of social support) | | | | XX |
| CTI Phase 3 (field-based service delivery, linkages to community supports, inclusion of social support) | | | | XX |
| **Summary Score** | | | | **YY** |
| Not  Implemented | Poorly  Implemented | Adequately Implemented | Well  Implemented | Ideally  Implemented |
| 1.0-1.4 | 1.5-2.4 | 2.5-3.4 | 3.5-4.4 | 4.5-5.0 |

*XX = average score across items within each domain, YY = average of domain items

4. Expert-rated Assessment Ratings
   - Strengths *(extracted from areas of adequate fidelity and field note narratives)*
   - Areas for Growth *(bullet points identifying domains of inadequate fidelity,* *acknowledging contextual factors found in field note narratives)*
   - Summary and recommendations *(summary of high priority areas to improve fidelity, hyperlinks to resources and tools to guide improvement)*

| **Fidelity Domain** | **Adequate Fidelity** | **Area for Growth** |
| --- | --- | --- |
| Clinical supervision in CTI practice | X |  |
| Case load size | X |  |
| Use of phases |  | X |
| Focus areas |  | X |
| Decreasing intensity of case management |  | X |
| Duration of case management (6 months, no early discharges) | X |  |
| Field-based delivery of care |  | X |
| Linkages to community-based services | X |  |
| Termination and transfer-of-care practices |  | X |

*X placed in one column per domain to characterize adequate versus inadequate fidelity
